# Diffuse alveolar hemorrhage caused by IgA deposition associated with multiple myeloma

**DOI:** 10.1002/ccr3.2151

**Published:** 2019-04-16

**Authors:** Atsuki Furube, Naho Kagiyama, Takashi Ishiguro, Yotaro Takaku, Kazuyoshi Kurashima, Yoshihiko Shimizu, Noboru Takayanagi

**Affiliations:** ^1^ Department of Respiratory Medicine Saitama Cardiovascular and Respiratory Center Saitama Japan; ^2^ Department of Diagnostic Pathology Saitama Cardiovascular and Respiratory Center Saitama Japan

**Keywords:** bronchoalveolar lavage, diffuse alveolar hemorrhage, multiple myeloma, transbronchial lung biopsy

## Abstract

We report a man with diffuse alveolar hemorrhage caused by multiple myeloma who was diagnosed with the aid of bronchoalveolar lavage and transbronchial lung biopsy. Multiple myeloma should be considered as an important differential diagnosis in patients with diffuse alveolar hemorrhage, and bronchoscopy may help to differentiate the cause.

## 1 INTRODUCTION

Many different diseases cause diffuse alveolar hemorrhage (DAH),^1^ and it needs to be accurately diagnosed early in its course. The available reports on patients with multiple myeloma (MM) and DAH state that the causes are pulmonary‐renal syndromes (PRS),^2^, ^3^, ^4^ immunoglobulin A (IgA) deposition in the alveolar wall,^5^, ^6^ certain drugs,^7^, ^8^, ^9^, ^10^, ^11^ postautologous stem cell transplantation,^12^ and uncertain origin.^13^ DAH was the initial symptom in three cases of PRS and in two cases of IgA deposition in the alveolar wall, and in other reports, DAH occurred during the course of MM treatment. Here, we report the third known case of DAH in a 77‐year‐old man with undiagnosed MM who was admitted to our hospital for treatment of DAH caused by deposition of IgA.

## 2 CASE REPORT

**Figure 1:**
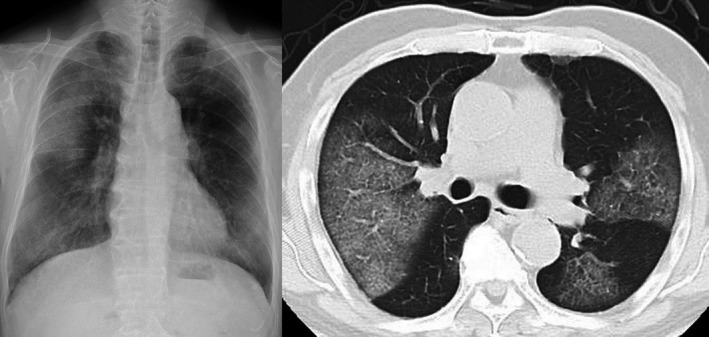
Chest X‐ray and computed tomography images showed bilateral ground‐glass opacities

**Figure 2:**
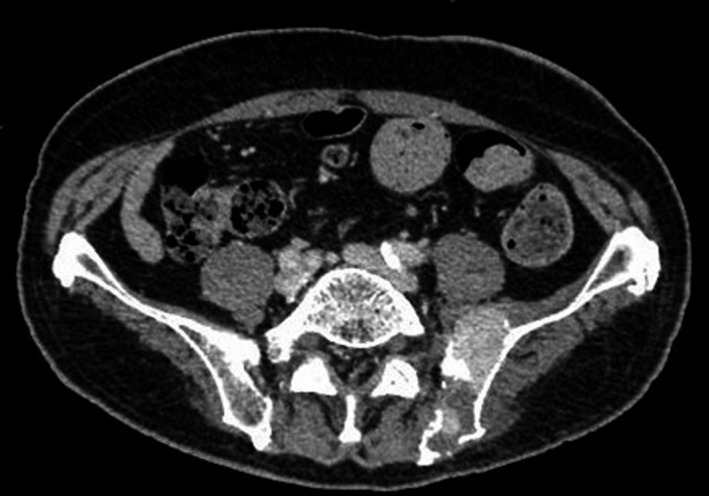
Abdominal computed tomography showed a left iliac tumor

**Figure 3:**
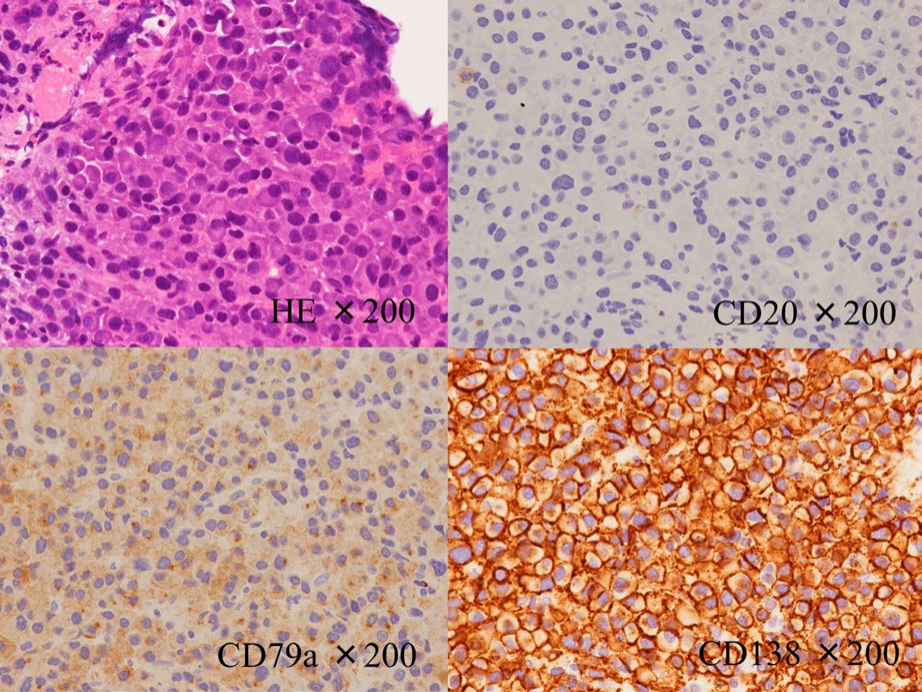
Histology of the left iliac tumor showed abnormal cells stained with hematoxylin and eosin. Immunostaining of the biopsied specimen showed the cells to be negative for CD20 but positive for CD79a and CD138, indicating that the abnormal cells were plasma cells

A 77‐year‐old man was admitted to our hospital with hemoptysis and slight fever of two weeks’ duration. He had been treated and followed for an old myocardial infarction and was taking clopidogrel and aspirin. On admission, his blood pressure was 111/55 mm Hg, pulse rate was 72/min, and body temperature was 37.2°C. His arterial oxygen saturation was 91% on O_2_ at 2 L/min via nasal canula, and arterial blood gas analysis showed a PaO_2_ of 61.9 Torr, PaCO_2_ of 30.3 Torr, and pH of 7.46. Chest X‐ray and computed tomography (CT) showed bilateral ground‐glass opacities (Figure 1). Blood tests revealed a white blood cell count of 10 900/µL (normal, 3900‐9800) (neutrophils 82.9%, lymphocytes 9.0%, eosinophils 2.3%, monocytes 5.7%), hemoglobin 10.7 g/dL (normal, 13.5‐17.6), platelets 202 000/µL (normal, 130 000‐369 000), prothrombin time 13.9 seconds (normal, 10.7‐12.9), activated partial thromboplastin time 49.4 seconds (normal, 24.0‐39.0), C‐reactive protein 14.3 mg/dL (normal, <0.3), LDH 386 IU/L (normal, 119‐229), total protein 7.4 g/dL (normal, 6.6‐8.4), serum albumin 3.2 g/dL (normal, 3.8‐5.2), serum creatinine 0.8 mg/dL (normal, 0.6‐1.1), blood urea nitrogen 16 mg/dL (normal, 8‐20), calcium 8.2 mg/dL (normal, 8.8‐10.5), IgA 946 mg/dL (normal, 110‐410), immunoglobulin G (IgG) 964 mg/dL (normal, 870‐1700), and immunoglobulin M (IgM) 37 mg/dL (normal, 35‐220). Urinalysis showed protein of 223 mg/dL (normal, <19) and was negative for occult blood. We initially suspected bacterial pneumonia, with antiplatelet agents as the cause of the DAH. Although we started antibiotics and stopped the antiplatelet agents, his pulmonary lesions gradually worsened, and hemoptysis continued on the 2nd hospital day. He required the administration of oxygen at an FiO_2_ of 80% to maintain an O_2_ saturation above 90%, which precluded the performance of bronchoscopy. We also considered systemic vasculitis and started a 3‐day course of intravenous methylprednisolone (1000 mg/d) followed by oral prednisolone at 40 mg/d. However, the corticosteroid therapy was not effective: the inflammatory reaction increased, and his respiratory function continued to deteriorate. Therefore, we began another 3‐day course of intravenous methylprednisolone on the 9th hospital day. Tests for antibodies associated with connective tissue diseases, including MPO‐ANCA, PR3‐ANCA, and anti‐GBM antibody, were all negative. Although his IgA value was high, his IgG and IgM values were normal. Serum electrophoresis showed no M protein, but serum immunoelectrophoresis revealed the presence of IgA‐κ‐type M protein. Serum free light chain (sFLC) assay showed a κ/λ ratio of 21.4 (normal, 0.2‐1.8). The patient’s β2‐microblobulin was 1.6 mg/L (normal, 0.9‐1.9), and he was negative for urine Bence Jones protein. An abdominal CT performed to search for MM revealed a left iliac tumor (Figure 2). A CT‐guided needle biopsy of this tumor was performed that revealed abnormal cells on hematoxylin and eosin staining. Immunostaining of the biopsied specimen showed the cells to be positive for CD79a and CD138, indicating that the abnormal cells were plasma cells (Figure 3). Moreover, immunostaining of these cells was positive for IgA and kappa light chain, and thus we diagnosed the patient as having IgA‐κ‐type MM.

**Figure 4:**
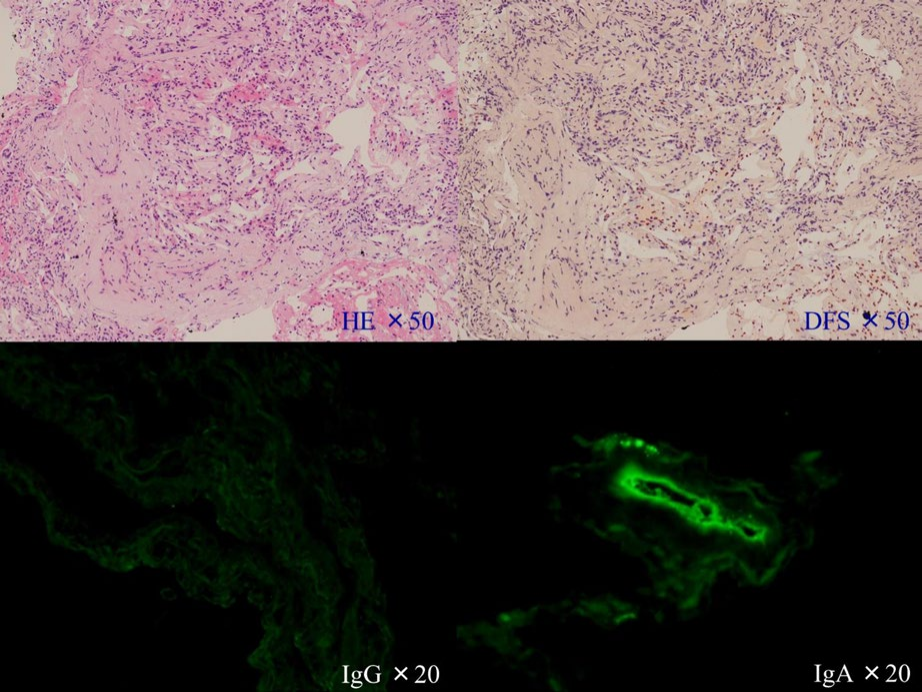
Transbronchial lung biopsy showed interstitial fibrosis negative for plasma cells by hematoxylin and eosin staining. We found no amyloid deposition in the lungs by direct fast scarlet stain. Immunofluorescence staining revealed deposition of IgA on a blood vessel wall, but no deposition of IgG was found

After the patient’s respiratory condition improved, bronchoalveolar lavage fluid (BALF) was obtained from the left B5 by bronchoscopy. We instilled 150 mL of 0.9% NaCl and aspirated 70 mL of bloody fluid containing 93.2% macrophages, 4.7% lymphocytes, 2.1% neutrophils, with a CD4/8 ratio of 0.5, and 25% hemosiderin‐laden macrophages. A transbronchial lung biopsy (TBLB) showed interstitial fibrosis but was negative for plasma cells. We found no amyloid deposition in the lung by direct fast scarlet staining. However, deposition of IgA was revealed on a blood vessel wall by immunofluorescence staining, but no deposition of IgG was present (Figure 4). We thus thought that the patient had DAH caused by IgA deposition due to MM of Durie/Salmon stage IIA and International Staging System stage II. We reduced the corticosteroid dose due to the diagnosis of MM, and he was discharged from our hospital on the 55th hospital day. Nine days later, treatment with lenalidomide and corticosteroid was started by the Department of Hematology in another hospital. His serum IgA and κ/λ ratio decreased, and the DAH improved after two courses of the treatment; however, the tumor in the left ilium did not improve.

## 3 DISCUSSION

This case showed that IgA deposition due to MM might cause DAH. We used the diagnostic criteria of the International Myeloma Working Group^14^ to diagnose MM in this patient based on the serum M protein level, extramedullary plasmacytoma, and presence of the iliac lesion. Other main conditions associated with MM, that is, renal disorder, hypercalcemia, and anemia, were not identified in this patient.

de Prost et al^1^ reported that one‐third of all occurrences of DAH were caused by immune diseases, especially vasculitis and antiglomerular basement membrane antibody syndrome, and connective tissue diseases. Among the non‐immune‐mediated etiologies, systolic or diastolic cardiac dysfunction of the left ventricle and valvular heart disease were the main causes, followed by infection and drug‐induced DAH. Cancer was identified in 4% of patients.^2^ In the present case, DAH was diagnosed on the basis of the clinical course and BALF and TBLB findings. Antibodies related to connective tissue diseases, including MPO‐ANCA and PR3‐ANCA, were all negative. Serum levels of IgA and IgA‐κ‐type M protein, the sFLC κ/λ ratio, and the iliac lesion helped us to diagnose MM.

Among five diagnosed cases of MM due to DAH, three were caused by PRS^2^, ^3^, ^4^ and two by IgA deposition in the alveolar wall,^5^, ^6^ in which IgA‐κ‐type M proteins were detected. Schreiber et al. reported that immunohistochemistry revealed dense pericapillary and perivascular deposits of IgA.^6^ In both cases, the treatment for MM affected the DAH. As in the present patient, immunofluorescence of a TBLB specimen from these two patients also showed deposition of IgA on a blood vessel wall. We thought perivascular deposits of IgA might have caused collapse of these vessel walls that led to DAH.

After treatment for MM was initiated in our patient, his serum IgA level and sFLC κ/λ ratio decreased, and his DAH and respiratory condition improved, although his iliac lesion remained unchanged. We are continuing to follow this patient to determine whether his MM has improved.

In conclusion, we reported a case of DAH caused by IgA deposition associated with MM. In this patient, TBLB revealed deposition of IgA in the alveolar wall. MM should be considered as one possible cause of DAH as the initial symptom of MM might well be DAH. IgA deposition in the alveolar wall should also be considered as a potential cause of DAH in the absence of PRS.

## CONFLICT OF INTEREST

None declared.

## AUTHOR CONTRIBUTIONS

AF: is the guarantor of the paper, taking responsibility for the integrity of the work as a whole, from inception to published article. NK, TI, YT, KK, YS, and NT: aggregated the data and helped draft the discussion of the manuscript.
